# Attention influences the effects of the previous form orientation on the current motion direction estimation

**DOI:** 10.1038/s41598-024-52069-5

**Published:** 2024-01-16

**Authors:** Si-Yu Wang, Xiu-Mei Gong, Lin-Zhe Zhan, Fan-Huan You, Qi Sun

**Affiliations:** 1 School of Psychology, Zhejiang Normal University, Jinhua, People’s Republic of China; 2 Intelligent Laboratory of Zhejiang Province in Mental Health and Crisis Intervention for Children and Adolescents, Jinhua, People’s Republic of China; 3 Key Laboratory of Intelligent Education Technology and Application of Zhejiang Province, Zhejiang Normal University, Jinhua, People’s Republic of China

**Keywords:** Psychology, Human behaviour

## Abstract

Recent studies have found that the estimates of motion directions are biased toward the previous form orientations, showing serial dependence, and the serial dependence does not involve cognitive abilities. In the current study, we conducted two experiments to investigate whether and how attention—a cognitive ability—affected the serial dependence. The results showed that serial dependence was present in the current study, reproducing the previous findings. Importantly, when the attentional load reduced the reliability (i.e., estimation accuracy and precision) of previous form orientations (Experiment 1), the serial dependence decreased, meaning that the biases of motion direction estimates toward previous form orientations were reduced; in contrast, when the attentional load reduced the reliability of current motion directions (Experiment 2), the serial dependence increased, meaning that the biases of motion direction estimates toward previous form orientations were increased. These trends were well consistent with the prediction of the Bayesian inference theory. Therefore, the current study revealed the involvement of attention in the serial dependence of current motion direction estimation on the previous form orientation, demonstrating that the serial dependence was cognitive and the attentional effect can be a Bayesian inference process, initially revealing its computational mechanism.

## Introduction

“*The wind has calmed, yet petals still do fall; Birds chirp, but mountains are secluded all.”*

Motion and stillness are two basic states of the world. Early researchers generally proposed that our visual system evolved ventral (“what”) and dorsal (“where”) pathways to process static and moving information, respectively^1–3^. However, our daily experiences remind us that they are closely linked. For example, a fast-moving object like a shooting star, can generate a static streak on the observer’s retina, named form; in contrast, streaks, such as those found in a comic book following a runner, can create a sense of motion. Recent studies support our intuition and find that the processing of form and motion are closely linked^4–10^.

**Figure 1:**
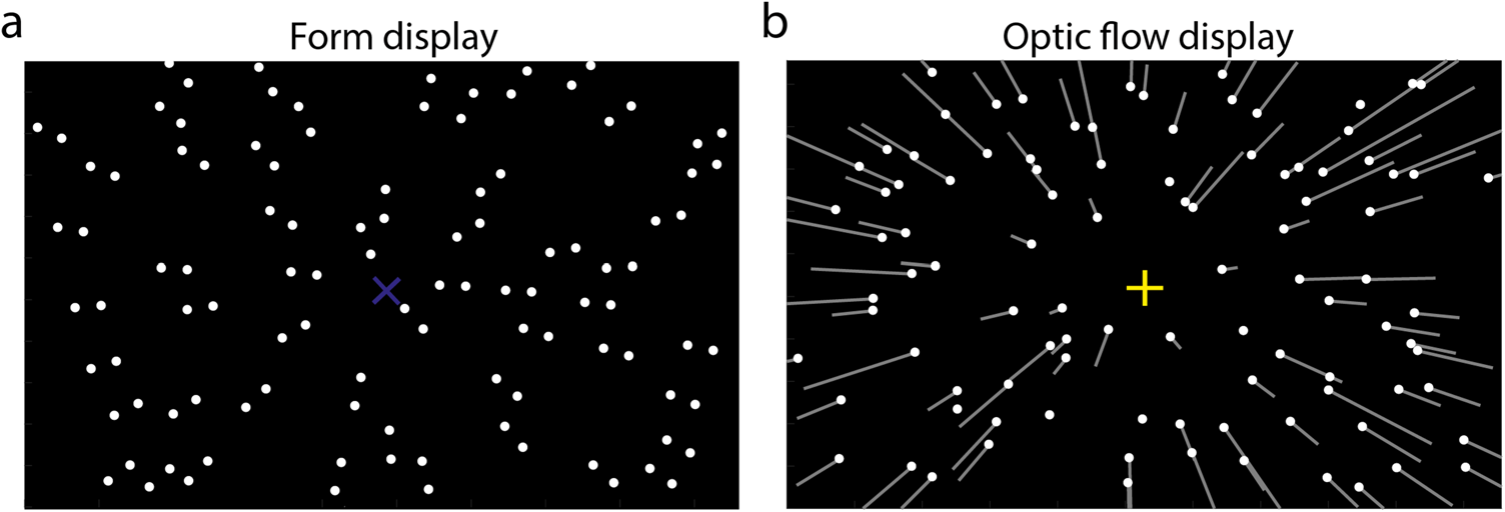
Illustrations of stimulus displays used in the current study. (**a**) Form display in which dots were paired. All dot pairs oriented toward one position of the display (form FoE), indicated by a purple “ ×”. (**b**) Optic flow display in which all dots randomly positioned and moved radially outward from one position of the display (flow FoE), indicated by a yellow “ +”. Neither “ ×” nor “ +” were visible in the experiment, so were the white lines.

In the studies mentioned above, form and motion features are presented simultaneously. For example, Niehorster et al.^11^ showed participants a series of Glass patterns^12^ consisting of dots. Half of the dots were paired with the other half of the dots, and the dot pairs were oriented toward one position on the display (Fig. 1a), generating a focus of expansion (form FoE) that indicated the form orientation. Meanwhile, all dots moved radially outward from one position on the display, generating an optic flow. It looked as if all dots emanated from that position (flow FoE), indicating the motion direction. They asked participants to report the motion direction and found that the perceived motion direction was biased toward the form orientation, suggesting an integration of the two features presented simultaneously (see also Refs.^4,7,8,13^).

In addition, previous studies have found that the estimates of physical features are systematically compressed toward the mean of the stimulus distribution, known as central tendency^14^, which occurs at the estimation of time interval^15–18^, loundness^19^, line length^20–22^, color^23,24^, facial expression^25,26^. These studies push us to ask whether the overall central tendency can be divided into one trial. That is, the central tendency is the sum of compression bias toward the previous trials. Fischer and Whitney^27^ first found that the orientation estimates were biased toward the previously seen orientations, showing a serial dependence^28–34^. Similar results have been observed for various features, such as spatial position^35,36^, motion direction^37–39^, facial features (e.g., expression, identity, and attractiveness)^40–42^, and numerosity^41,43^. Researchers generally agree that serial dependence reflects the integration of information across the temporal domain, and its main role is to help observers maintain perceptual continuity (see Refs.^44,45^ for reviews). Inspired by these studies, Wang et al.^34^ presented form and optic flow displays sequentially. They found that the estimate of the current motion direction was biased toward the previously seen form orientation, showing serial dependence (also see Refs.^46,47^). These studies revealed the integration of the two features across the temporal domain.

Aside from examining the range of features that show serial dependence, researchers also investigate its occurrence mechanisms. Is serial dependence a purely information-driven process, or are some cognitive abilities (e.g., working memory, attention) also involved in serial dependence? Fischer and Whitney^27^ varied the positions of stimuli across trials. They found a strong serial dependence when the stimuli were presented in the same position (also see Refs.^41,48,49^), suggesting that attention affected serial dependence. In addition, Bliss et al.^35^ varied the interval durations between the stimulus and response displays to manipulate the involvement degree of working memory. As the interval increased, working memory became more involved in perception. They found that if participants reported the perceived orientation immediately after the stimulus (i.e., the interval is 0 ms), the orientation estimate of the current trial was biased away from the previously seen orientations, showing a negative aftereffect. In contrast, when the interval was increased (e.g., 1 s, 3 s, 6 s, 10 s), the orientation estimate of the current trial was biased toward the previously seen orientations, showing an attractive serial dependence. Hence, they concluded that working memory was involved in the serial dependence^35,38,50^. Bae and Luck^50^ successfully decoded the features of previous trials from the EEG data in the intervals of current trials, supporting the involvement of working memory in serial dependence (also see Ref.^38^).

Wang et al.^34^ adopted a dual-task paradigm to examine whether working memory was involved in the serial dependence between form and flow features. Specifically, they asked participants to finish three blocks of trials. Each block corresponded to one condition. In the form-absent condition, only one flow display was presented; in the form-present condition, an extra form display was presented, but participants were only asked to fixate on the display; in the form-memory condition, participants were asked to remember the form orientation and report the form orientation after responding to the motion direction. Therefore, form orientation would take more working memory resources in the form-memory condition than in the form-present condition. They found that the sizes of serial dependence were not significantly different between form-present and form-memory conditions. They, therefore, concluded that working memory was not involved in the serial dependence of the motion direction estimation on the previously seen form orientation.

It has been demonstrated that working memory is closely linked with attention. The features captured by attention were more easily stored in working memory^51–53^, and the features stored in working memory affected the deployment of attention^54–56^. In addition, the two cognitive abilities shared some common neural basis^44,57,58^. Although Wang et al.^34^ found working memory was not in the serial dependence of the motion direction estimation on the previously seen form orientation, it remained unclear whether attention affected the serial dependence, which was our first question.

Moreover, some previous studies on serial dependence proposed that it was consistent with Bayesian inference theory^28,31,39^. Specifically, when the reliability of current features was weakened, observers would rely more on the previously presented feature to estimate the current feature. It has been known that attention directly affects the activities of neurons. When one feature captures more attentional resources, the neurons responding to the feature become more excited^59–62^. That is, the feature representation will be more reliable. Therefore, if attention affects the serial dependence of the motion direction estimation on the previously seen form orientation, the effects can be consistent with the Bayesian inference theory.

In summary, in the current study, we design two experiments to investigate whether attention affects the serial dependence of the motion direction estimation on the previously seen form orientation and whether the effects are consistent with the Bayesian inference theory. Addressing these questions can clarify the processing mechanism underlying the serial dependence, enriching the previous studies. Additionally, it can enhance our understanding of the cognitive and computational mechanisms underlying the cross-temporal integration of form orientations and motion directions.

## Results

### Experiment 1 attentional load on the previous form display

**Figure 2:**
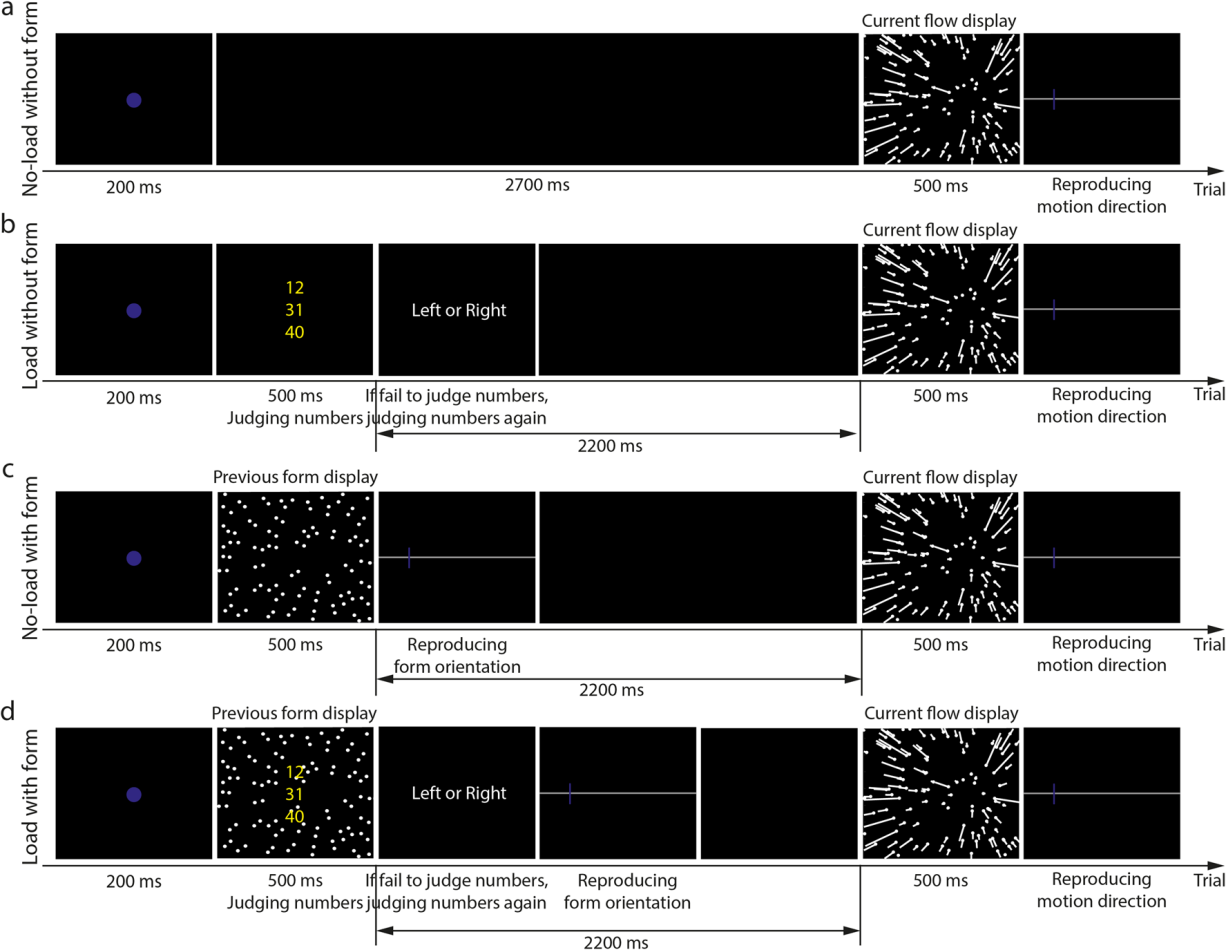
Illustrations of trial procedures of four conditions in Experiment 1.

Experiment 1 aimed to examine whether attention affected the serial dependence of the motion direction estimation from optic flow (Fig. 1b, see “Methods” for details) on the previously seen form orientation (Fig. 1a). Participants were asked to finish four blocks of trials, each block corresponding to one condition: no-load without form (Fig. 2a), load without form (Fig. 2b), no-load with form (Fig. 2c), and load with form (Fig. 2d) conditions. Note that in the load conditions, participants were asked to sum the first two integers and compare the sum with the last integer (number-addition task) before reporting the form orientation estimates.

We first examined whether the attentional load affected the estimation accuracy (estimation error) and precision (standard deviation) of the estimation of form orientations and motion directions. Then, we calculated the residual estimation error of motion directions in the load and no-load conditions. Specifically, the residual estimation error in the no-load condition meant the difference in estimation error of motion directions between no-load with form and no-load without form conditions; so as in the load conditions. To examine whether attentional load affected the serial dependence, we compared the difference in the residual estimation error between the load and no-load conditions.

#### Estimation error and standard deviation

**Figure 3:**
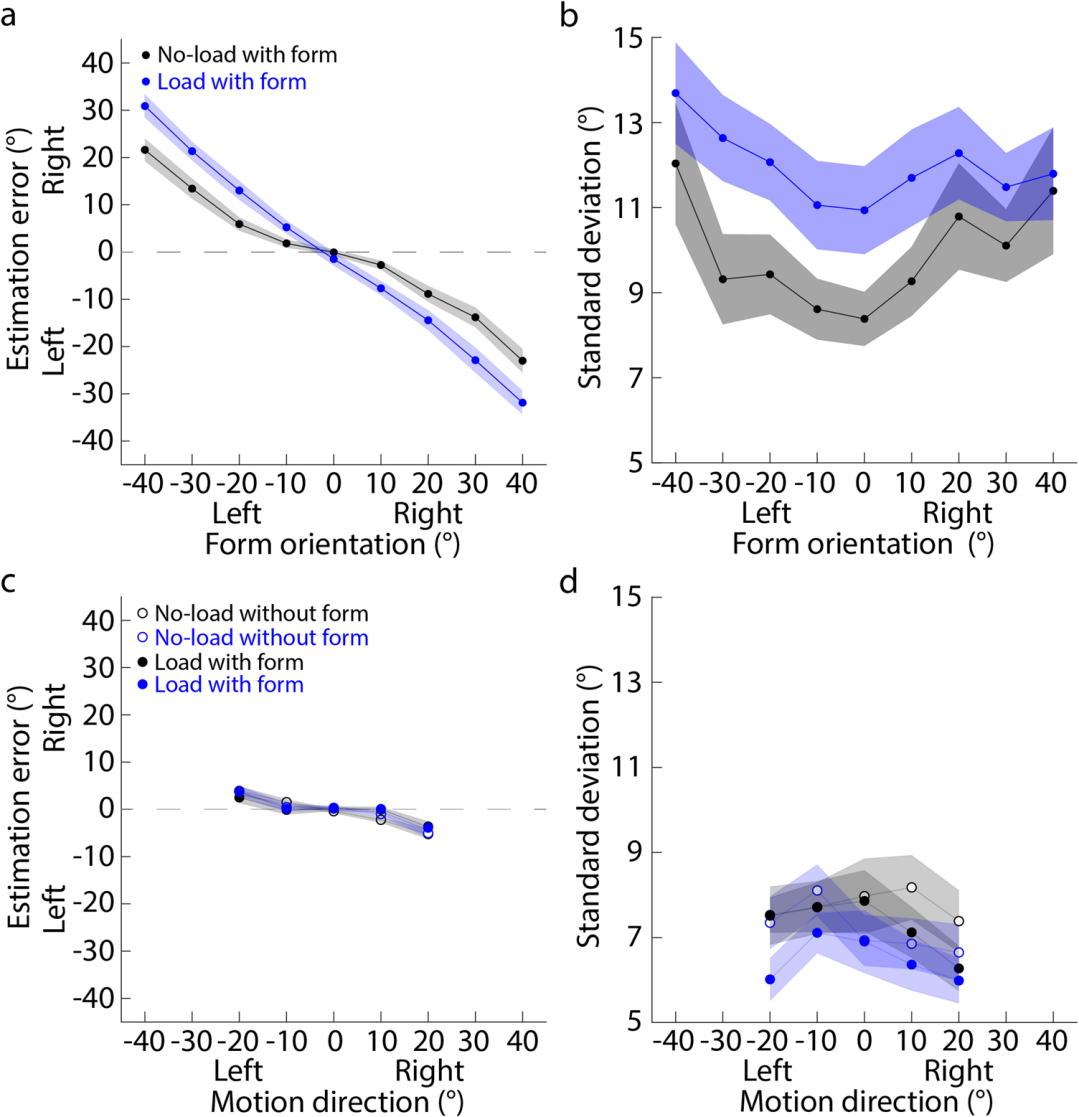
Overall estimation performance of Experiment 1. (**a**) and (**b**) Results of form orientation estimation. (**c**) and (**d**) Results of motion direction estimation. (**a**) and (**c**) Estimation error is against the actual form orientation or motion direction. (**b**) and (**d**) Standard deviation is against the actual form orientation or motion direction. Each dot indicates the mean estimation error or standard deviation averaged across all participants. Left and right on the x-axis mean that the form orientation or motion direction is to the left or right of the display center (0°); left and right on the y-axis of (**a**) and (**c**) mean that the estimate is to the left or right of the actual value. Shaded area indicates the standard error across all participants.

Figure 3a and b plot the estimation error and standard deviation against the actual form orientations. It clearly shows that both the estimation error and standard deviation increased when the attentional load is added in the form orientation estimation task (blue dots in Fig. 3a and b). In addition, the signs of estimation errors are opposite to actual form orientations, indicating center bias in the form orientation estimation. 2 (load conditions: no-load vs. load) × 9 (form orientations) repeated measures ANOVA showed that for the estimation error, the interaction effect between two factors was significant (Greenhouse–Geisser corrected: *F* (1.61, 30.53) = 19.48, *p* < 0.001, partial *η*^2^ = 0.51). Further simple effect with Newman-Keuls correction showed that the estimation errors of ± 40°, ± 30°, ± 20°, and ± 10° were significantly larger in the load condition than in the no-load condition (*p* < 0.040), suggesting that the attentional load reduced the accuracy of the form orientation estimation, showing an increased center bias in the estimation of the form orientation. In addition, the standard deviation in the load condition (Mean ± SE: 11.96 ± 0.85) tended to be larger than that in the no-load condition (9.93 ± 0.89) (*F* (1.00, 19.00) = 3.33, *p* = 0.84, partial *η*^2^ = 0.15), suggesting that the attentional load reduced the precision of the form orientation estimation.

Figure 3c and d plot the estimation error and standard deviation against the actual motion direction. The estimation errors and standard deviations of four conditions are croweded together. However, it is clear that the signs of the estimation errors are opposite to the signs of motion directions, suggesting center bias is in the motion direction estimation. 2 (form conditions: without vs. with form) × 2 (load conditions: no-load vs. load) × 5 (motion directions) repeated-measures ANOVA showed that for the estimation error, neither the main effect of load conditions nor the main effect of flow conditions was significant (Greenhouse–Geisser corrected: *F* (1.00, 19.00) = 0.48, *p* = 0.50, partial *η*^2^ = 0.024; *F* (1.00, 19.00) = 0.96, *p* = 0.34, partial *η*^2^ = 0.048). Only the interaction between form conditions and motion directions was significant (*F* (1.49, 28.35) = 4.89, *p* = 0.023, partial *η*^2^ = 0.21). Further simple effect with Newman-Keuls correction showed that when motion directions were + 10° and + 20°, the estimation errors in the without-form condition (0.012 ± 0.64, − 1.60 ± 0.46) were significantly smaller than those in the with-form condition (− 3.76 ± 1.01, − 5.14 ± 0.87) (*p*s < 0.019). These suggested that the attentional load in the previous form orientation estimation task did not affect the accuracy and center bias of the motion direction estimation. However, the previously presented form might reduce the estimation accuracy and increase center bias. In addition, the standard deviation in the no-load condition (7.53 ± 0.49) was significantly larger than that in the load condition (6.83 ± 0.45) (*F* (1.00, 19.00) = 13.33, *p* = 0.0017, partial *η*^2^ = 0.41), and the standard deviation in the with-form condition (7.47 ± 0.51) also tended to be significantly larger than that in the without-form condition (6.89 ± 0.45) (*F* (1.00, 19.00) = 4.28, *p* = 0.052, partial *η*^2^ = 0.18). Together, these results suggested that both the attentional load and the previous form display reduced the precision of the motion direction estimation.

#### Serial dependence

**Figure 4:**
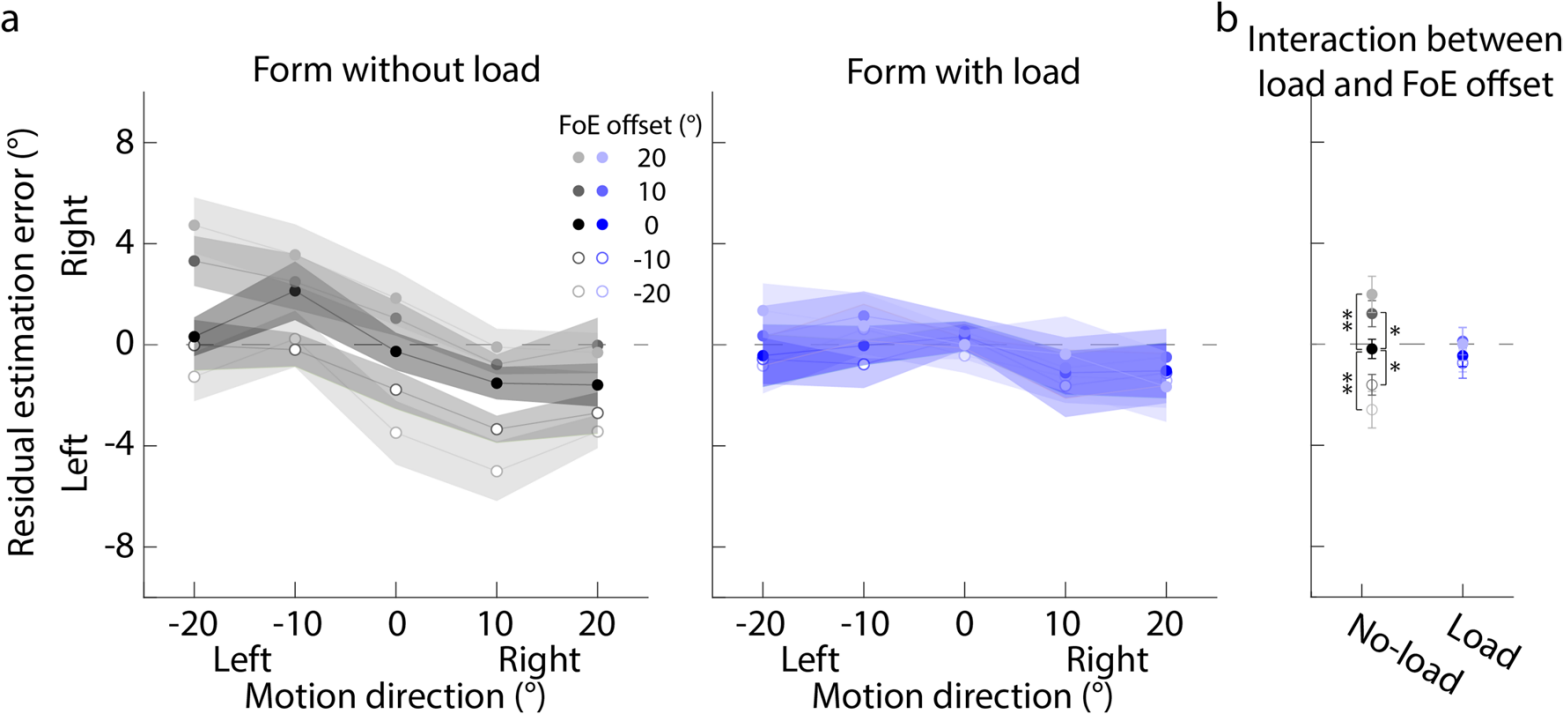
Residual estimation error against the motion direction of Experiment 1. (**a**) Left and right panels correspond to the no-load and load conditions. Each dot indicates the mean residual estimation error averaged across all participants. Left and right on the y-axis mean that the motion direction estimate in the with-form condition is to the left or right of that in the without-form condition. (**b**) The interaction effect between load conditions and FoE offsets. Each dot indicates the mean residual estimation error averaged across five motion directions. Error bar indicates the standard error. *p < 0.05; **p < 0.01; ***p < 0.001.

Figure 4a plots the residual estimation error against the motion direction. It clearly shows that in the no-load condition (left panel), when the form orientation is to the right of the motion direction (positive FoE offsets), the residual estimation error, on average, tends to be positive (gray solid dot in Fig. 4b), suggesting that the motion direction estimation in the with-form condition is to the right of that in the without-form condition. The trend is reversed when the form orientation is to the left of the motion direction (negative FoE offsets; gray circles in Fig. 4b). In contrast, when the attentional load is added in the form orientation estimation task, the above trends seem to be disappeared (right panel in Fig. 4a, blue dots in Fig. 4b).

A 2 (load conditions: no-load vs. load) × 5 (FoE offsets) × 5 (motion directions) repeated measures ANOVA showed that the interaction between load conditions and FoE offsets was significant (Greenhouse–Geisser corrected: *F* (1.68, 31.85) = 6.96, *p* < 0.001, partial *η*^2^ = 0.70). Simple effect analysis with Newman-Keuls correction showed that in the no-load condition (gray dots in Fig. 4b), the estimate of the motion direction was significantly biased toward the right when the form orientation was to the right of the motion direction, and vice versa; in contrast, there was no significant difference between FoE offsets in the load condition (blue dots in Fig. 4b). Therefore, the results suggested that the estimate of the motion direction was biased toward the previous form orientation, showing an attractive serial dependence, and attention affected the attractive serial dependence.

#### Summary

To sum up, the current experiment found that the estimation of motion directions was biased toward the previously seen form orientations, showing an attractive serial dependence, consistent with previous studies^34^. In addition, the attractive serial dependence was affected by attention, providing further evidence for the claims that attention was involved in serial dependence^27,41,48,49,63^ and serial dependence was post-perceptual^28,32,35^.

Importantly, the current experiment revealed that the serial dependence between form and flow features could be consistent with the Bayesian inference theory^64–66^. The Bayesian inference theory proposes that observers optimally combine multiple features to perceive one feature. When one feature’s reliability decreases, observers will rely more on the other features. Previous neurophysiological studies have found that the activities of neurons selectively responding to one feature increase with the attentional resources allocated to that feature^60–62,67^. When the attentional resources were distracted by a number-addition task, the internal representation of the form orientation became less precise (supported by our standard deviation results, Fig. 3b). That is, the form orientation was unreliable. Hence, the attractive serial dependence was significantly reduced (Fig. 4a), well matching the prediction of the Bayesian inference theory.

Additionally, readers may notice that the estimation error of the 40° form display is close to − 30° (Fig. 3a), meaning that the response distribution for the form FoE spans approximately − 10 to 10 degrees. This can indicate that the previous response remains highly similar across FoE offset conditions (Fig. 4b). As a result, any potential impact of form FoE may be obscured if the serial dependence is consistently directed toward the prior response, irrespective of the actual strength of dependency. To test the proposal, we analyzed the correlation between the estimation errors of form and flow FoEs. The results showed that on individual level, half of participants’ correlations (10/20 participants) were not significant; and on the group level, the correlation was also insignificant (*p* = 0.28). These suggested that the serial dependence was barely biased toward the previous response. In addition, the response probe would be randomly re-positioned on the response line after participants’ each response, which can somewhat inhibit the response bias.

### Experiment 2 attentional load on the current optic flow display

**Figure 5:**
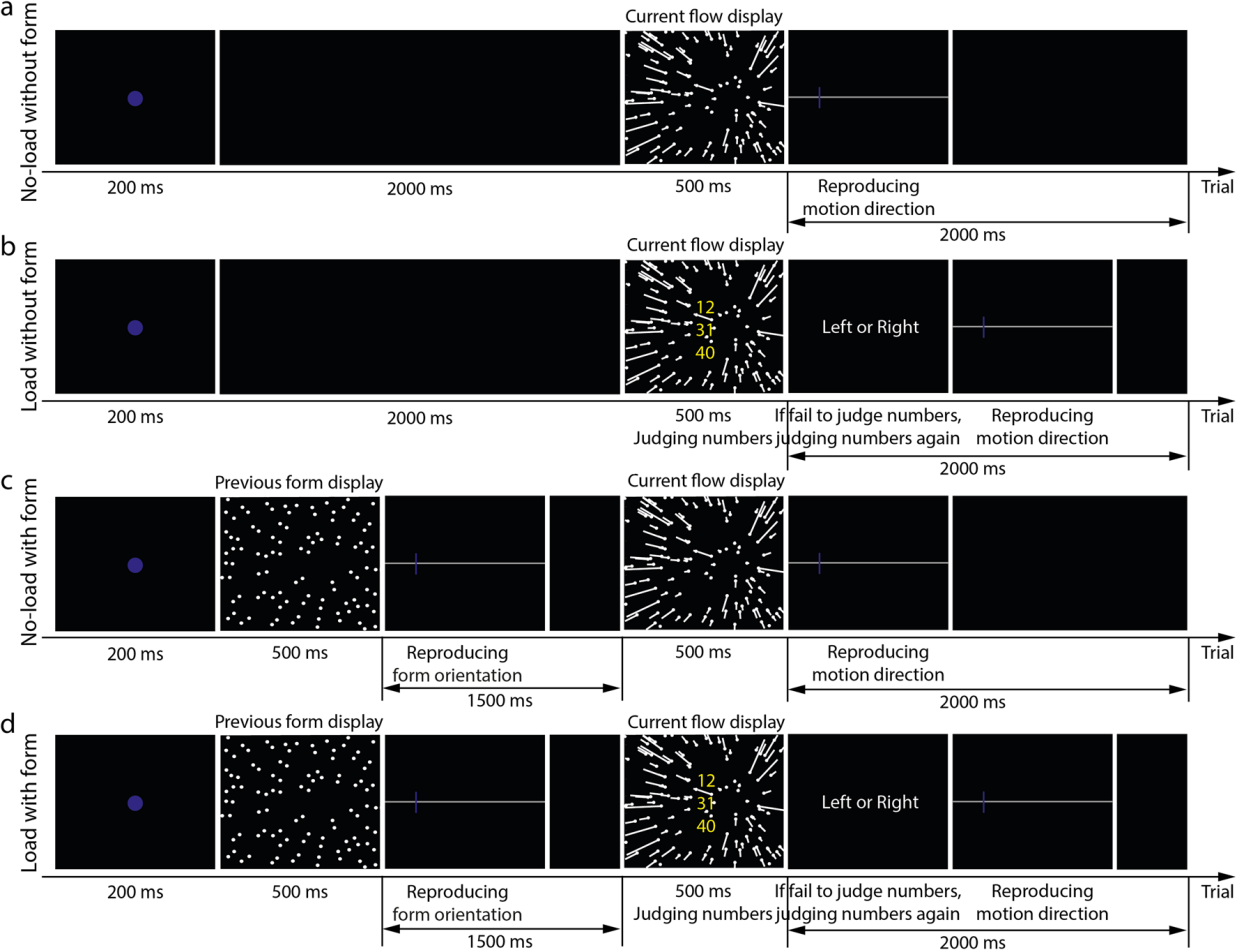
Illustrations of trial procedures of four conditions in Experiment 2.

Experiment 1 found that attention affected the serial dependence of the motion direction estimation on previous form orientation. The effect well matched the prediction of the Bayesian inference theory^64–66^. According to the Bayesian inference theory, as long as the reliability of one feature decreases, observers will increase the reliance on the other features, which is a bidirectional process. However, Experiment 1 reduced the reliability of previous form displays by distracting the attention resources. Hence, Experiment 1 only revealed that the serial dependence of the motion direction estimation on previous form orientation was affected by the reliability of previous feature, indicating a partial Bayesian inference process. In Experiment 2, we added attentional load to the motion direction estimation task (Fig. 5b and d) to reduce the reliability of flow displays and investigated whether the attractive serial dependence increased in this situation (See "Methods" for more details). If so, then the serial dependence of the motion direction estimation on previous form orientation was a Bayesian inference process.

#### Estimation error and standard deviation

**Figure 6:**
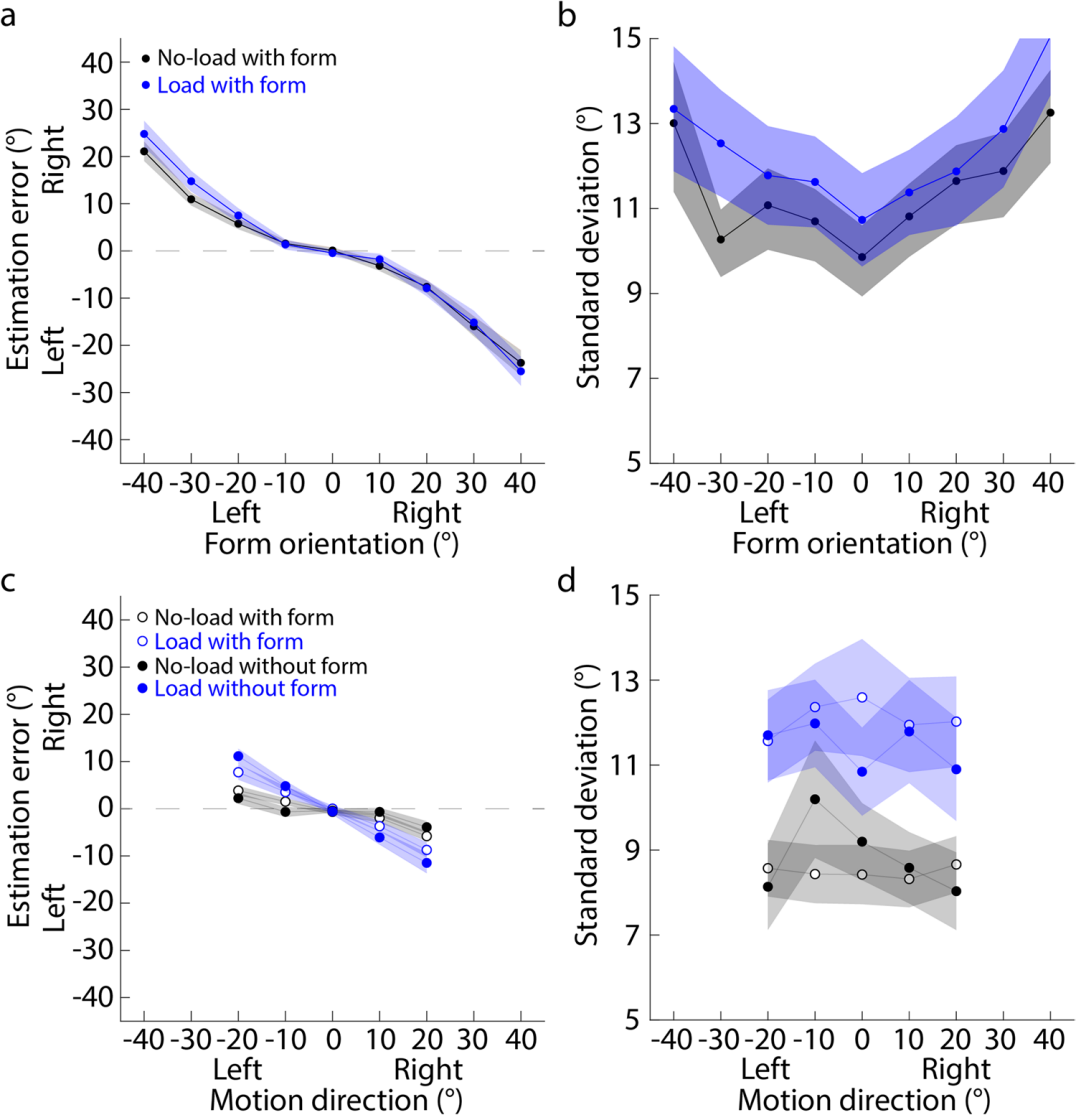
Overall estimation performance of Experiment 1. (**a**) and (**b**) Results of form orientation estimation. (**c**) and (**d**) Results of motion direction estimation. (**a**) and (**c**) Estimation error is against the actual form orientation or motion direction. (**b**) and (**d**) Standard deviation is against the actual form orientation or motion direction. Each dot indicates the mean estimation error or standard deviation averaged across all participants. Left and right on the x-axis mean that the form orientation or motion direction is to the left or right of the display center (0°); left and right on the y-axis of (**a**) and (**c**) mean that the estimate is to the left or right of the actual value. Shaded area indicates the standard error across all participants.

In the current experiment, the attentional load was presented on the optic flow displays that were shown after the form displays. Figure 6a and b show the results of the form orientation estimation. They clearly show that neither the estimation errors nor the standard deviations show significant difference between the no-load and load conditions. In addition, like Experiment 1, the signs of estimation errors are opposite to actual form orientations, indicating center bias in the form orientation estimation. 2 (load conditions: no-load vs. load) × 9 (form orientations) repeated measures ANOVA showed that regardless of the estimation error or standard deviation, neither the main effect of load conditions nor the interaction effect between two factors was significant (Greenhouse–Geisser corrected: estimation error, *F* (1.00, 19.00) = 3.44, *p* = 0.079, partial *η*^2^ = 0.15; *F* (2.56, 48.67) = 1.82, *p* = 0.16, partial *η*^2^ = 0.087; standard deviation, *F* (1.00, 19.00) = 1.07, *p* = 0.31, partial *η*^2^ = 0.053; *F* (3.83, 72.82) = 0.58, *p* = 0.67, partial *η*^2^ = 0.030), suggesting that the attentional load presented after the form display did not affect the accuracy and precision of the form orientation estimation.

Figure 6c and d show the results of the motion direction estimation. It clearly shows that when the attentional load is added to the motion direction estimation, both the estimation error and standard deviation increase (blue dots in two figures). In addition, the signs of estimation errors are opposite to the signs of motion direction, hence center bias is in the estimation of motion directions and increases with the increasing attentional load. 2 (form conditions: without vs. with form) × 2 (load conditions: no-load vs. load) × 5 (motion directions) repeated-measures ANOVA showed that for the estimation error, the interaction between load conditions and motion directions was significant (*F* (1.22, 23.08) = 16.91, *p* < 0.001, partial *η*^2^ = 0.47). Further simple effect with Newman-Keuls correction showed that when motion directions were ± 10° and ± 20°, the estimation errors in the no-load condition were significantly smaller than those in the load condition (*p*s < 0.0095), suggesting that the attentional load reduced the accuracy of the estimation of motion directions. In addition, the standard deviation in the load condition (11.77 ± 0.94) was significantly larger than that in the no-load condition (8.66 ± 0.65) (*F* (1.00, 19.00) = 14.37, *p* < 0.001, partial *η*^2^ = 0.431), suggesting that the attentional load reduced the precision of the motion direction estimation.

#### Serial dependence

**Figure 7:**
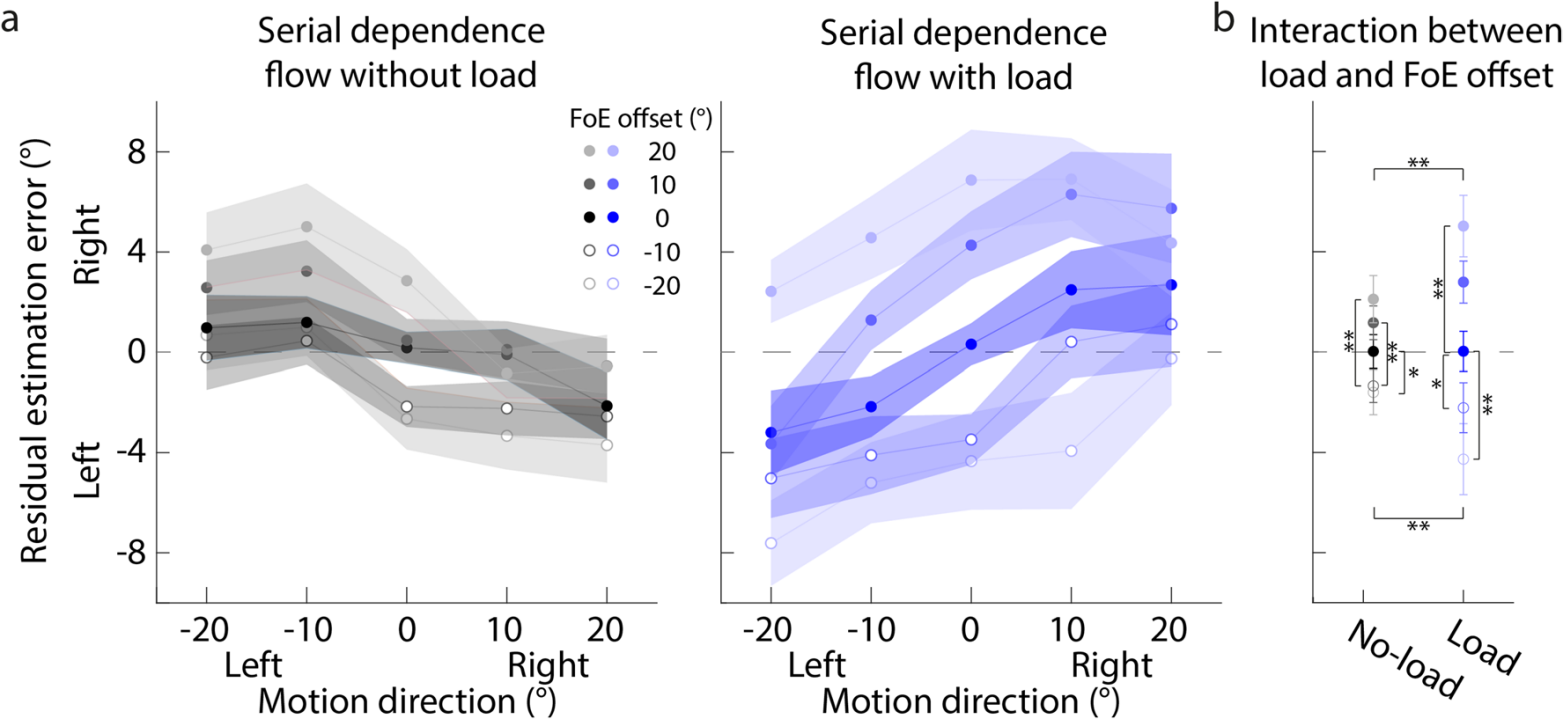
Residual estimation error against the motion direction of Experiment 2. (**a**) Left and right panels correspond to the no-load and load conditions. Each dot indicates the mean residual estimation error averaged across all participants. Left and right on the y-axis mean that the motion direction estimate in the with-form condition is to the left or right of that in the without-form condition. (**b**) The interaction effect between load conditions and FoE offsets. Each dot indicates the mean residual estimation error averaged across five motion directions. Error bar indicates the standard error. *p < 0.05; **p < 0.01; ***p < 0.001.

Figure 7a plots the residual estimation error against the motion direction. It clearly shows that in the no-load condition (left panel), when the form orientation is to the right of the motion direction (positive FoE offsets), the residual estimation error, on average, tends to be positive (gray solid dot in Fig. 7b), suggesting that the motion direction estimation in the with-form condition is to the right of that in the without-form condition. The trend is reversed when the form orientation is to the left of the motion direction (negative FoE offsets; gray circles in Fig. 7b). More importantly, when the attentional load is added in the motion direction estimation task, the above trends seem to be larger (right panel in Fig. 7a, blue dots in Fig. 7b).

A 2 (load conditions: no-load vs. load) × 5 (FoE offsets) × 5 (motion directions) repeated measures ANOVA showed that the main effect of FoE offsets was significant (Greenhouse–Geisser corrected: *F* (1.23, 23.43) = 20.55, *p* < 0.001, partial *η*^2^ = 0.52). Post-hoc analysis with Newman-Keuls correction showed that compared to the estimates of motion directions when the FoE offsets were 0° (i.e., the form orientations were equal to the motion directions), the estimates of motion directions were significantly more biased toward to the right when the FoE offsets were + 10° and + 20° (i.e., the form orientations were to the right of the motion directions) (*p*s < 0.021), and vice versa (*p*s < 0.033). This suggested that the current estimation of motion directions relied on the previous form orientation, showing an attractive serial dependence. Importantly, the interaction between FoE offsets and load conditions was significant (Greenhouse–Geisser corrected: *F* (1.59, 30.11) = 7.06, *p* = 0.0053, partial *η*^2^ = 0.27). Simple effect analysis with Newman-Keuls correction (Fig. 7b) showed that when the FoE offsets were ± 20°, the residual estimation error in the load condition was significantly larger than that in the no-load condition (*p*s < 0.0047), suggesting that when the attentional load was added in the motion direction estimation task, observer increased their reliance on the previous form orientation. Therefore, the results suggested that the estimate of motion direction was biased toward the previous form orientation, showing an attractive serial dependence, and attention affected the attractive serial dependence, consistent with Experiment 1.

#### Summary

To sum up, Experiment 2 well reproduced the findings of Experiment 1. Specifically, the estimation of motion direction was biased toward the previously seen form orientation, showing an attractive serial dependence, which was affected by attention.

Importantly, the current experiment found that when the reliability of current flow displays was reduced, participants increased their reliance on the previously seen form display to estimate the motion direction. That is, the serial dependence of the motion direction estimation on previous form orientation was affected by the reliability of current flow displays. Together with the finding that the serial dependence of the motion direction estimation on previous form orientation was affected by the reliability of previous form display (Experiment 1), we concluded that the serial dependence of the motion direction estimation on previous form orientation was consistent with the Bayesian inference theory.

In addition, Fig. 7 showed that on average, in the no-load condition, the residual error decreased with increasing motion direction; whereas in the load condition, the residual error increased with increasing motion direction. This trend suggested that the estimates of motion directions in the load condition were more overestimated than those in the no-load condition. As shown in Fig. 6d, the attentional load reduced the precision of the internal representation of motion displays, meaning that the internal noise was increased. Wei and Stocker (2015, 2017)^68,69^ developed a Bayesian inference model constrained by efficient coding, which found that when the internal noise of physical features increased, the features tended to be overestimated. Therefore, the trend could imply that observers encoded the motion direction efficiently.

## Discussion

The results of two experiments in the current study showed that attention affected the serial dependence of the motion directions estimation on the previous form orientations. It is known that attention is a cognitive ability. Therefore, the results suggested that serial dependence was cognitive (i.e., some studies used post-perceptual), enriching the previous studies^41,48,49,63^. Importantly, the results also showed that the attentional load could reduce the reliability of motion or form displays. Especially, when attentional load reduced the reliability of previous form displays, the size of the serial dependence was decreased, meaning that the motion direction estimates were less biased toward the previous form orientation; in contrast, when the attentional load reduced the reliability of current optic flow displays, the size of the serial dependence was increased, meaning that the motion direction estimates were more biased toward the previous form orientation. These trends were well consistent with the predictions of Bayesian inference theory^64–66^, which proposed that when the reliability of one feature was reduced, observers would rely more on other physical features (e.g., prior) to estimate. Therefore, the influence of attention on the serial dependence of the motion directions estimation on the previous form orientations can be a Bayesian inference process.

The most instructive finding in the current study is the attentional effect on the serial dependence of current motion direction estimation on the previous form orientation. Previous studies on serial dependence generally changed the position of the focus of attention (e.g., Ref.^27^), or the contents in attention (e.g., Ref.^63^), or asked participants not to report on some trials (e.g., Refs.^32,70^). The attentional load paradigm used in the current study provided a new method for examining the effects of attention on serial dependence, which was developed based on the attentional load theory (ALT)^59,60,71–73^. The ALT posits that the attentional resources are limited. When one task takes some attentional resources, the attentional resources deployed to other tasks must be reduced. As a result, the task performance will be impaired^71,74–77^ or even the internal neural activities are inhibited^59–61,67^. Accordingly, we directly manipulated the deployment of attentional resources and revealed the effect of attention on serial dependence, providing researchers with new methods for future studies.

When the attentional resources allocated to the estimation task of form orientations (motion directions) were reduced, the reliabilities (estimation accuracy and precision) of form (motion) displays were reduced as well (Figs. 3a,b, 6c,d). The bias of motion direction estimates toward form orientations would be reduced (increased), consistent with the predictions of the Bayesian inference theory^64–66^. Previous studies have proposed that serial dependence can be a Bayesian inference process. However, these studies only found that the serial dependence was affected by the reliability of current features independent of the reliability of previous features, which is a partial Bayesian inference process^28,30,31^. In contrast, our results showed a bidirectional Bayesian inference process. We proposed that the different processes could be due to different occurrence stages of stimulus reliabilities. Specifically, the previous studies have varied the stimulus reliabilities on the sensory level. For example, Ceylan et al.^28^ directly increased the spatial frequency of oriented Gabors to increase the stimulus reliability, which is a feedforward process. However, in our study, the stimulus reliability was manipulated by varying the attentional resources, which is a feed-backward process. These differences imply that the Bayesian inference processes may differ at the sensory and cognitive levels, an open question for future studies.

Moreover, the current study directly revealed the involvement of attention in self-motion direction (i.e., heading) perception from optic flow, supporting the notion that heading perception was information driven (i.e., perceptual). Previous studies have demonstrated that observers can accurately estimate heading direction by locating the flow FoE^78,79^ and early proposed that the estimation was purely information driven^27,29,80,81^. However, several recent studies showed that post-perceptual cognitive abilities were in heading estimation from optic flow^39,51,82^. For example, Sun et al.^82^ found that heading estimates were affected by past experiences (i.e., heading distributions); Sun et al.^38^ revealed the involvement of working memory in the heading estimation by successfully decoding previous headings from the EEG data of current trials. The current study explicitly showed the involvement of attention. These studies, together, clarify the cognitive nature of heading estimation from optic flow. Meanwhile, future studies can explicitly investigate whether and how other cognitive abilities (e.g., decision-making, long-term memory) affect heading estimation and explore the network structures among these abilities.

Furthermore, previous studies found asymmetric integrations between form orientations and motion directions that were presented simultaneously. That is, the bias of motion direction estimates toward form orientations differed from that of form orientation estimates toward motion directions^7,9^. The current study only examined the across-temporal integration of current motion directions with previous form orientations. It remained unclear whether our findings were also observed in the across-temporal integration of current form orientation with previous motion directions. Revealing the mechanisms of the simultaneous and across-temporal integrations between two features will depict comprehensive structures of the processing of the two features. Besides, several studies have found that the estimates of current motion directions were biased toward the responses of previous trials but biased away from the actual features of previous trials^33,46^. The current study did not differentiate the effects of previous response and features on the current perception, leaving an open question for future studies. Xu et al.^39^ found that the previous responses did not affect the motion direction estimation of current trials, which adopted the similar motion displays that were more complex than those in Refs.^33,46^. If future studies with complex motion and form displays showed different result patterns, then it could be proposed that the dissociation of serial dependences on the response and stimulus could be modulated by the complexity of stimuli.

It is known that several different theories have been proposed to explain the effects of attention on visual perception. Some propose that attention opens the gate for the relevant information but filters the irrelevant information early in the sensory information processing stream, known as the early selective view^83^; but some propose that attention occurs at the later (higher) stage of information processing, known as the late selective view^84^. According to the distinct views, attention can occur at the perceptual and post-perceptual stages. Due to the simultaneously presented attentional load and form display in the current study, we are unable to identify the occurrence stages of attention on the serial dependence. Additionally, the current study showed form and optic flow sequentially, with a time interval between two stimuli. Sun et al.^38^ found that previously presented optic flow (i.e., prior knowledge) was first stored in working memory and then integrated with the currently presented optic flow. Their experimental procedures were similar to the load with form condition in the current study (Fig. 2c), which prompted us to propose that the previously presented optic flow was also stored in working memory. Future studies can be designed to solve these questions. Moreover, previous studies have debated a lot on the occurrence stages (perceptual vs. post-perceptual) of serial dependence (see a review discussion in Ref.^45^). Answering the aforementioned questions may provide more evidence to solve the controversy.

In summary, the current study systematically showed that attention affected the across-temporal integration between form orientations and motion directions and the effects could be a Bayesian inference process. These findings enhanced our understanding about the cognitive and computational mechanisms underlying the serial dependence between different features.

## Methods

### Participants

The experiment was approved by the Scientific and Ethical Review Committee of the Department of Psychology at Zhejiang Normal University. We obtained all participants’ written informed consent form before starting the experiment. All aspects of data collection and analysis were conducted in accordance with guidelines approved by the Committee for the Protection of Human Subjects in Zhejiang Normal University. The sample size was decided based on previous studies (e.g., Refs.^39,85^). All participants (Experiment 1: 8 males, 12 females; 18–26 years old; Experiment 2: 8 males, 12 females; 18–25 years old) were enrolled from our university. All participants had normal or corrected-to-normal vision and were naive to the purpose of the experiment.

### Stimuli and apparatus

The current experiment included two types of stimulus displays: a static form display and a dynamic optic flow display. Both displays (112° H × 80° V; luminance: 0.24 cd/cm^2^, Fig. 1) consisted of 90 dots (diameter: 0.24°; luminance: 22.5 cd/cm^2^). The dots in the form display (Fig. 1a) were paired. The distance between two dots was 1°. All dot pairs were oriented to one position of the display, generating a focus of expansion (FoE, purple cross in Fig. 1a), named form FoE indicating the form orientation. The optic flow display simulated observers moving in a 3D dot-cloud (depth range: 0.2–10 m) at a speed of 1 m/s, which generated a dynamic motion display (Fig. 1b). It looked as if all dots emanated from a position of the display (yellow fixation in Fig. 1b), called flow FoE indicating the motion direction.

The motion direction (i.e., flow FoE) was randomly selected from the ± 20°, ± 10°, and 0°. Negative and positive values indicate that the motion direction is to the left or right of the display center (i.e., the straight-ahead direction). The form orientation was to the left or right of the motion direction by 0°, 10°, or 20°, named as FoE offsets. For example, when the motion direction was − 20°, then the form orientation could be − 40°, − 30°, − 20°, − 10° and 0°.

On some trials, three integers (RGB: [0, 0, 200]; 1.76° V × 1.76° H) were presented vertically on the form or optic flow display center. The gap between the two numbers was 0.44°. The first two integers were randomly selected from the range [11, 40] while the last integer was randomly selected from the range [40, 92].

The experiment was programmed using MATLAB with the Psychophysics Toolbox 3. Stimuli were displayed on a 27-inch ASUS monitor (resolution: 2560 H × 1440 V pixels; refresh rate: 60 Hz) with an NVIDIA GeForce GTX 1660Ti graphics card.

### Procedures

All participants were seated in front of a computer monitor in a light-excluded room. The viewing distance was 20 cm. They viewed the display monocularly with their right eye to reduce the conflict between the motion parallax and binocular disparity depth cues. Their heads were fixed by a chinrest. During the experiment, they were asked to fixate on the display center. They were not allowed to move their eyes, head, and body to reduce the effects of non-optic flow information on the heading estimation.

Each participant of the two experiments was asked to finish four blocks, each corresponding to one condition (Figs. 2 and 5): no-load without form condition, load without form condition, no-load with form condition, and load with form condition.

#### Experiment 1

Each trial in the no-load without form condition (Fig. 2a) started with a 200-ms fixation, followed by a 2.7-s blank display. A 500-ms current optic flow display was then presented, followed by a response display. Participants were asked to report their motion direction estimates by moving a mouse-controlled probe along a horizontal line. The next trial started immediately after the participant’s response.

The trial procedure in the load without form condition (Fig. 2b) was similar to that in the no-load without form condition, except that the 2.7-s blank display was replaced with a number addition task, in which three integers were presented on the display center for 500 ms. Participants were asked to sum the first two integers up and compare the sum to the last integer as quickly and accurately as possible. If they failed to respond in the 500-ms integer display, a number reminder display was presented in which they were reminded to respond to the number addition task. After their response, a blank display was presented. The total duration of the integer, number reminder, and blank displays was fixed at 2.7 s.

The trial procedure in the no-load with form condition (Fig. 2c) was similar to that in the load without form condition, except that the 500-ms integer display was replaced with a form display, named as the previous form display. The reminder display was replaced with a form orientation response display in which a probe and a horizontal line were presented. Participants were asked to move a probe to report their form orientation estimates. After the response, a blank display was presented. The duration of the previous form display, response display, and blank display was fixed at 2.7 s.

The trial procedure in the load with form condition (Fig. 2d) was the combination of the trial procedures in the load without form condition and the no-load with form condition.

The no-load and load without form conditions included 5 current motion directions (± 20°, ± 10°, and 0°), each of which was repeated 50 times. Thus, there were 250 trials (5 current motion directions × 50 trials) in each condition. In no-load and load with form conditions, each current heading was accompanied by 5 FoE offsets—the difference between the previous form orientation and current motion direction. Each FoE offset was repeated 10 times, resulting in a total of 250 trials (5 current motion directions × 5 FoE offsets × 10 trials). Note that the 250-trial condition blocks were the ideal case. If participants failed to respond to the number addition task or the heading estimation task within the fixed time, the trial would be added back to the trial list again. As a result, some participants would finish more than 250 trials in one condition block.

Before each condition block, participants were given approximately 15 practice trials, randomly selected from the block, to familiarize them with the condition. After the practice, the corresponding block started, lasting for about 20 min. The conducting sequences of the four conditions were counterbalanced across participants.

#### Experiment 2

The design of Experiment 2 was similar to Experiment 1, except that (1) in the load (with and without form) conditions, the attentional load was presented on the current flow display (Fig. 5b and d). Therefore, participants were asked to first conduct the number addition task when the flow display was presented, like Experiment 1, with a 500-ms completion time limit. If they failed, then a reminder display was presented. After judging the numbers, they reported their motion direction estimate by moving a mouse-controlled probe on a horizontal line, followed by a blank display. The total durations of the reminder display, motion direction response display and the blank display were fixed at 2 s. (2) In the with-form conditions (Fig. 5c and d), participants were asked to report their form orientation estimate immediately after the form orientation display, followed by a blank display. The total durations of the response display and blank display were fixed at 1.5 s. Please see Fig. 5 for more details.

### Data analysis

The data analysis methods were the same in the two experiments. In the load (without and with form) conditions, we first calculated the accuracy of the number addition task. If the accuracy of one participant was below 0.75, then the participant would be removed. The results showed that all participants’ accuracies were beyond 0.75.

Next, we removed the trials in which participants did not finish the number addition task or the form orientation estimation task within the specified time interval. Thus, each participant had 250 trials in each condition block.

Participants’ estimates for motion directions and form orientations were collected. Firstly, for form and optic flow displays, we used the estimate to minus the actual value, getting the estimation error. The larger the value of estimation errors, the lower the estimation accuracy. In addition, if the sign of the estimation error is opposite to the sign of the actual value, then center bias is in the estimation. The larger the estimation error, the stronger the center bias. Secondly, we also calculated the standard deviation of estimates for each actual value. The larger the standard deviation, the lower the estimation precision. For form displays, a two-factors (load conditions × form orientations) repeated-measures ANOVA was conducted on the estimation error and standard deviation to examine whether the attentional load affected the overall performance of form orientation estimation. For optic flow displays, a three-factors (form conditions × load conditions × motion directions) repeated-measures ANOVA was conducted on the estimation error and standard deviation to examine whether the attentional load and previously presented form affect the overall performance of motion direction estimation.

The above procedure also generated the errors of the motion direction estimates in the four conditions (no-load without form, no-load with form, load without form, load with form). Then, the error in the no-load without form condition was subtracted by the error in the no-load with form condition, getting the residual estimation error (REE). The REE was proposed to be induced by the previous form orientation when all attentional resources were focused on the processing of the form orientation (REE_no-load_). In addition, we used the error in the load with form condition to subtract the error in the load without form condition, getting another REE. The REE was proposed to be induced by the previous form orientation when attention was distracted by the number addition task (REE_load_). i.e., the attentional resources on the form orientation processing were reduced. A three-factors (load conditions × flow orientations × motion directions) repeated-measures ANOVA was conducted on the REE to examine whether the estimation of the current motion direction was biased by the previous form orientation and whether the bias was modulated by the attentional load.

## Data Availability

All relevant data are available from Qi Sun (sunqi_psy@zjnu.edu.cn). This study was not preregistered.
